# Dentin Bond Integrity of Filled and Unfilled Resin Adhesive Enhanced with Silica Nanoparticles—An SEM, EDX, Micro-Raman, FTIR and Micro-Tensile Bond Strength Study

**DOI:** 10.3390/polym13071093

**Published:** 2021-03-30

**Authors:** Aasem M. Alhenaki, Esra A. Attar, Abdullah Alshahrani, Imran Farooq, Fahim Vohra, Tariq Abduljabbar

**Affiliations:** 1 Department of Prosthetic Dental Sciences, College of Dentistry, King Saud University, Riyadh 11545, Saudi Arabia; aalhenaki@ksu.edu.sa (A.M.A.); asalshahrani@ksu.edu.sa (A.A.); fvohra@ksu.edu.sa (F.V.); 2 Oral and Maxillofacial Prosthodontics Department, Faculty of Dentistry, King Abdulaziz University, Jeddah 22252, Saudi Arabia; eaattar@kau.edu.sa; 3 Faculty of Dentistry, University of Toronto, Toronto, ON M5G 1G6, Canada; imran.farooq@mail.utoronto.ca

**Keywords:** dentin, silica, adhesive resin, bond strength, SEM-EDX, FTIR, micro-Raman

## Abstract

The objective of this study was to synthesize and assess unfilled and filled (silica nanoparticles) dentin adhesive polymer. Methods encompassing scanning electron microscopy (SEM)—namely, energy dispersive X-ray spectroscopy (EDX), micro-tensile bond strength (µTBS) test, Fourier transform infrared (FTIR), and micro-Raman spectroscopy—were utilized to investigate Si particles’ shape and incorporation, dentin bond toughness, degree of conversion (DC), and adhesive–dentin interaction. The Si particles were incorporated in the experimental adhesive (EA) at 0, 5, 10, and 15 wt. % to yield Si-EA-0% (negative control group), Si-EA-5%, Si-EA-10%, and Si-EA-15% groups, respectively. Teeth were set to form bonded samples using adhesives in four groups for µTBS testing, with and without aging. Si particles were spherical shaped and resin tags having standard penetrations were detected on SEM micrographs. The EDX analysis confirmed the occurrence of Si in the adhesive groups (maximum in the Si-EA-15% group). Micro-Raman spectroscopy revealed the presence of characteristic peaks at 638, 802, and 1300 cm^−1^ for the Si particles. The µTBS test revealed the highest mean values for Si-EA-15% followed by Si-EA-10%. The greatest DC was appreciated for the control group trailed by the Si-EA-5% group. The addition of Si particles of 15 and 10 wt. % in dentin adhesive showed improved bond strength. The addition of 15 wt. % resulted in a bond strength that was superior to all other groups. The Si-EA-15% group demonstrated acceptable DC, suitable dentin interaction, and resin tag formation.

## 1. Introduction

Dental resin composite is one of the most widely used restorative materials, exceeding 166 million restorations completed in the United States (U.S.) solely [1]. The clinical success of composite restoration depends on the properties of the dental adhesive [2]. Both dentin and enamel are diverse compositionally, and bond to resin composite material using different mechanisms [3]. Dentin is more organic as compared with enamel, and hence bonding to wet dentin is the most perplexing task in dentistry [4]. In the past, various studies have demonstrated that bonding to dentin results in a weaker bond strength of the restorative material [5,6]. Numerous elements play their part to improve the bonding properties. One important factor is the presence of an inorganic filler that can strengthen the mechanical properties of the polymer matrix [7]. The inclusion of filler nanoparticles can also enhance the durability of dental composite resins [8]. Researchers have previously added different fillers in the adhesives to study their mechanical properties and bond strength. These fillers included calcium-rich zeolite [9], hydroxyapatite (HA) [10], and graphene oxide (GO) [11]. These studies have revealed that the incorporation of inorganic fillers leads to an improvement of mechanical properties of the adhesive, remineralization potential, and increased bond strength [9,10,11].

Silica (Si) is one such filler that could increase the bondability of the material [12] and improves the bond strength of the adhesive [13]. The use of Si has risen recently in dentistry, and they are currently being used as fillers in glass-ionomer cement (GIC) and resin composites [14,15]. Si fillers can decrease polymerization shrinkage and enhance the mechanical properties of adhesives [15]. Previously, Guo et al. demonstrated that a combination of zirconia-Si could improve the fracture toughness and flexural strength of dental composites [16]. Bai et al. also reported that zinc doped Si nanoparticle fillers improved the antibacterial and mechanical properties (flexural strength and modulus, hardness, and compressive strength) of dental resin composites [17]. Timpe et al. in an earlier study revealed that incorporation of Si nanoparticles can improve the mechanical properties of dental composites; however, larger-sized particles could result in particle agglomeration and weakened mechanical properties [18]. Considering the beneficial properties of Si inclusion in the adhesive, it was decided to investigate the effect of various concentrations of Si in the experimental adhesive (EA).

The null hypothesis (H_o_) of our study was that the Si-containing adhesive would perform the same as the adhesive without Si (EA) in terms of the mechanical and physical properties of the adhesive, represented by micro-tensile bond strength (µTBS) and degree of conversion (DC). The present study aimed to integrate Si particles at different concentrations to an EA and analyze the bond strength, durability, and interactions of these adhesives with dentin, utilizing various analytical techniques including scanning electron microscopy (SEM)—energy dispersive X-ray spectroscopy (EDX), µTBS test, Fourier Transform Infrared (FTIR) spectroscopy, and DC analysis.

## 2. Materials and Methods

### 2.1. Synthesis of EA

The EA was produced using the methods earlier described by AlFawaz et al. [11]. Briefly, a mixture of different monomers that consisted of bisphenol A glycol dimethacrylate (BisGMA), triethylene glycol dimethacrylate (TEGDMA), 2-hydroxyethyl methacrylate (HEMA), and ethyl 4-dimethylamino benzoate and camphorquinone (Esstech Inc., Essington, PA, USA) were utilized. The adhesive used in our study was composed of w/w 50% BisGMA, 25% TEGDMA and 25% HEMA with a solvent (ethanol 30% m/m). In the monomer moles, we also incorporated n/n 0.5% each ethyl 4-dimethylamino benzoate and camphorquinone photo-initiators. Further, to act as an electron initiator, n/n 1.0% diphenyliodonium hexafluorophosphate (DPIHP) was added to the EA. This mixture was mixed utilizing a magnetic stirrer and synthesized inside a three-necked flask and a condenser (SA300; Sansyo, Tokyo, Japan). To inhibit photo-polymerization, we stored this adhesive mix in a secluded dark chamber that was shielded with a foil.

### 2.2. Procurement and Incorporation of Si Particles in EA

Si particles were commercially acquired (monodisperse silicon dioxide, non-porous silica—Sigma Aldrich, Mineapolis, MN, USA). The Si particles were added to 2 mL of ethanol in a micro vial, and at 37 °C the blend was sonicated for 10 min using an ultrasonicator (VWR USC-TH sonicator bath, Tokyo, Japan). The control group consisted of only EA without Si particles (unfilled adhesive, Si-EA-0%). Si 5%, 10%, and 15% particles were added to EA to yield three experimental groups, Si-EA-5%, Si-EA-10%, Si-EA-15%, respectively. To maximize the uniform distribution of Si particles, these were at first added to the resin and then sonicated for 10 min using an ultrasonic bath (VWR USC-TH sonicator bath, Tokyo, Japan). This step was followed by making the blend unvaried in an ultrasonic homogenizer (Q500 Sonica, Newtown, CT, USA) at pulse start/stop for 60 s at room temperature. Re-homogenization of the mixture was carried out after every use to warrant that the particles have homogenously disseminated in the adhesive. To ensure that the accurate amount of Si particles were added to the EA, these particles were weighed in milligrams, and their volume was calculated in milliliters. The following formula was utilized to compute *w/v* % for the three experimental groups, as recommended earlier by AlFawaz et al. [11]:

(1) $$w/v\;\%\;=\;\frac{\mathrm{Weight}\;\mathrm{of}\;\mathrm{the}\;\mathrm{solute}}{\mathrm{Volume}\;\mathrm{of}\;\mathrm{the}\;\mathrm{solution}\;}\;\times \;100$$

All the adhesives of our study were also stored at 4 °C and consumed within two weeks after synthesis.

### 2.3. Characterization of Si Particles

Scanning electron microscopy (SEM) was employed to observe the shape of Si pre-mixing in the adhesive. The Si particles were mounted on aluminium stubs and coated with a gold layer inside a sputter coating machine (Baltec SCD sputter, Scotia, NY, USA) for 2 min. For the characterization, an SEM (JEOL, JSM-6513, SEM, Tokyo, Japan) was utilized at an accelerating voltage of 30 kV. Various magnifications based on convenience were used to observe the morphology of Si particles.

Micro-Raman spectroscopy was also performed to characterize silica powder particles. A micro-Raman spectrophotometer (ProRaman-L Analyzer; TSI, Shoreview, MN, USA) with software (Raman reader^®^) was utilized to obtain Raman spectra(s). The laser beam was secured via a 0.9 objective lens and 600 mW power. A 60 s scan was performed three times for the silica powder. The specifics of the spectra were acquired using a laser beam wavelength of 532 nm between 600 and 1300 cm^−1^ with noise filtration.

### 2.4. Preparation of Tooth Samples

One hundred extracted maxillary premolar teeth (N = 100) were collected from the orthodontic clinic of the institute. Teeth that were caries-free and devoid of any other defects were included after cleaning them with an ultrasonic scaler (Superior Instruments Co., New York, NY, USA). Disinfection of the teeth was carried out first by using a disinfectant (Merck, Germany) followed by their storage in distilled water at 4 °C. By means of acrylic resin (Opti-Cryl, Columbia, SC, USA), these teeth were then embedded vertically in polyvinyl tubes of 4 mm diameter at the level of the cervical margin (enamel and cementum junction). To uncover the dentin tissue, the teeth occlusal enamel were cut using a water-cooled diamond saw (Buehler Isomet 2000 Precision saw, Lake Bluff, IL, USA). In the middle of the exposed dentin surface, a sound dentin area of ~5 mm was recognized and etched with 36% phosphoric acid (DeTrey conditioner, Dentsply, PA, USA) for 10 s. The etching was trailed by distilled water washing for 1 min and drying with cotton pellets. These one hundred prepared teeth were then randomly separated into four groups (each receiving 25 teeth, *n* = 25) and treated with their respective adhesives; gp-1: teeth treated with Si-EA-0%, gp-2: teeth treated with Si-EA-5%, gp-3: teeth treated with Si-EA-10%, and gp-4: teeth treated with Si-EA-15%. The respective adhesives were initially placed on a mixing pad, and the dentin surfaces were then treated with the adhesive for 15 s with the assistance of a micro-brush. This step was shadowed by air thinning for 5 s. The application of the adhesive was then repeated in a similar way for all the tooth samples. Photo-polymerization of all the specimens was then carried out using a light-curing device (Curing Light Eliphar S10; 3M ESPE, St. Paul, MN, USA) for 20 s from a distance of 10mm. These adhesive applied tooth samples were then sealed off with the help of resin composite (Filtek Supreme; 3M ESPE, St. Paul, MN, USA). The sealing was carried out by placing 2mm increments (not exceeding 4mm in height) with the help of an acrylic jig, a plastic instrument, and a condenser. Excessive material was detached, and 20 sec light curing of the interface was performed from all sides. Bonded tooth samples were stored in distilled water for a maximum of seven days at 37 °C.

### 2.5. µ TBS Analysis

Eighty teeth (twenty from each group) were used for µTBS analysis. From the twenty bonded teeth, ten teeth from each group were aged using thermocycling at 5 and 55 °C in distilled water baths (THE-1100, SD Mechatronik GmbH, Feldkirchen-Westerham, Germany). A total of 10,000 cycles for 30 s with a dwell time of 5 s were used for aging. The other ten bonded teeth remained non-aged and were kept safe in distilled water for one day, pre-sectioning. The sectioning of specimens belonging to each adhesive group was carried out to form beams of 1 mm × 1 mm of composite resin-adhesive with the help of a water-cooled diamond saw (Buehler Isomet 2000 Precision saw, IL, USA). In every tooth, six beams were formed (sixty beams in total), and for each group, five beams were analyzed for μTBS analysis.

### 2.6. Investigation of Bonded Adhesive–Dentin Interface Using SEM Spectroscopy

The remaining five bonded teeth from every group were partitioned using a slow speed saw (Buehler Isomet 2000 Precision saw, Lake Bluff, IL, USA) in order to create beams of 1 mm × 1 mm. The bonded adhesive–resin interface was observed using SEM-EDX spectroscopy. Employing a polisher (Beuhler Polisher, Lake Bluff, IL, USA), beams were wet polished, and placed in an ultrasonic bath containing distilled water (Bandelin Digital-Sigma-Aldrich Darmstadt, Germany) for 5 min. For the samples’ conditioning, 36% phosphoric acid (DeTrey conditioner, Dentsply, PA, USA) was applied. This was followed by washing and immersion in 5.25% sodium hypochlorite solution for 15 min. The samples were cleaned with distilled water, and then their dehydration was carried out with 80–100% concentrations of ethanol solution. The specimens were sputter coated with gold (as mentioned previously). An SEM (JEOL, JSM-6513, SEM, Tokyo, Japan) was used to observe samples’ adhesive–dentin interfaces with 30 kV voltage using different magnifications. EDX spectroscopy was also implemented to analyze the elemental distribution and presence of Si nanoparticles in the adhesives.

### 2.7. Degree of Conversion Analysis

Fourier transform infrared (FTIR) spectroscopy was utilized for DC analysis of all the adhesive groups, and all the samples were analyzed pre- and post-polymerization. Potassium bromide discs of the spectroscope (Shimadzu, Kyoto, Japan) were used for adhering to the adhesives. The absorbance peaks of carbon-carbon (C-C) double bonds were observed for uncured resin at the time when the adhesives continued to be in connection with FTIR sensors (Thermo Scientific Nicolet iS20 FTIR spectrometer, Waltham, MA, USA). The adhesives were cured for 40 s using a curing light and FTIR spectra were recorded again to detect the peaks. Using an already recognized technique [19], the aromatic C-C characteristic peaks (1607 cm^−1^) and aliphatic C=C absorbance peaks (1638 cm^−1^) were collected. To define the DC of our adhesives, FTIR spectra were perceived between 400 and 4000 cm^−1^. To compute conversion rates, ratios of (C-C and C=C) absorbance strengths (% of unreacted double bonds) pre-and post-polymerization were calculated using the formula described earlier [10] as follows:

DC = [1 − (Caliphatic/Caromatic)/(Ualiphatic/Uaromatic)] × 100%,(2)

where

C_aliphatic_ = 1638 cm^−1^ absorption peak of cured resin;
C_aromatic_ = 1607 cm^−1^ absorption peak of cured resin;
U_aliphatic_ = 1638 cm^−1^ absorption peak of uncured resin;
U_aromatic_ = 1607 cm^−1^ absorption peak of uncured resin.

### 2.8. Statistical Analysis

The µTBS and DC analysis results of the present study were statistically evaluated using SPSS-20.0 (IBM, Chicago, IL, USA). The normality of the data was first checked via the Kolmogorov–Smirnov test. Two-way ANOVA and Tukey’s post hoc multiple comparisons test. A *p*-value of <0.01 was considered statistically significant.

## 3. Results

### 3.1. SEM-EDX Analysis Results

**Figure 1:**
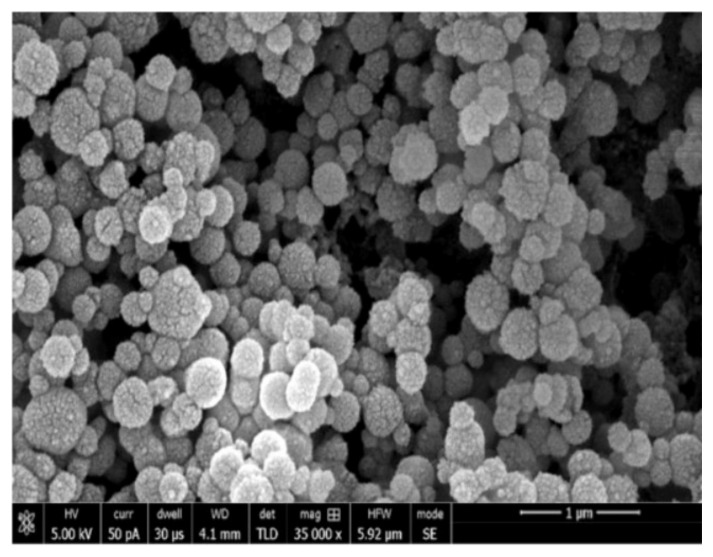
Presenting SiO2 non-porous nanoparticles (150 nm) spherical in shape appear to be in agglomerated and isolated form.

**Figure 2:**
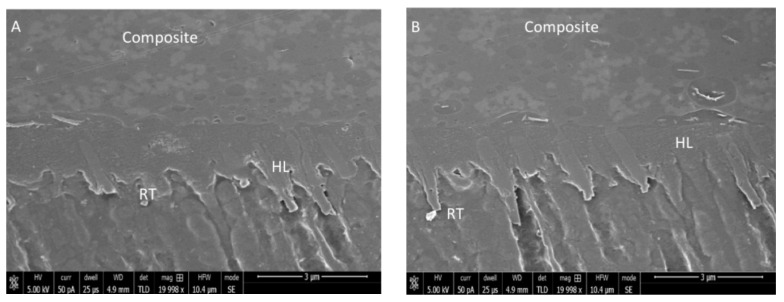

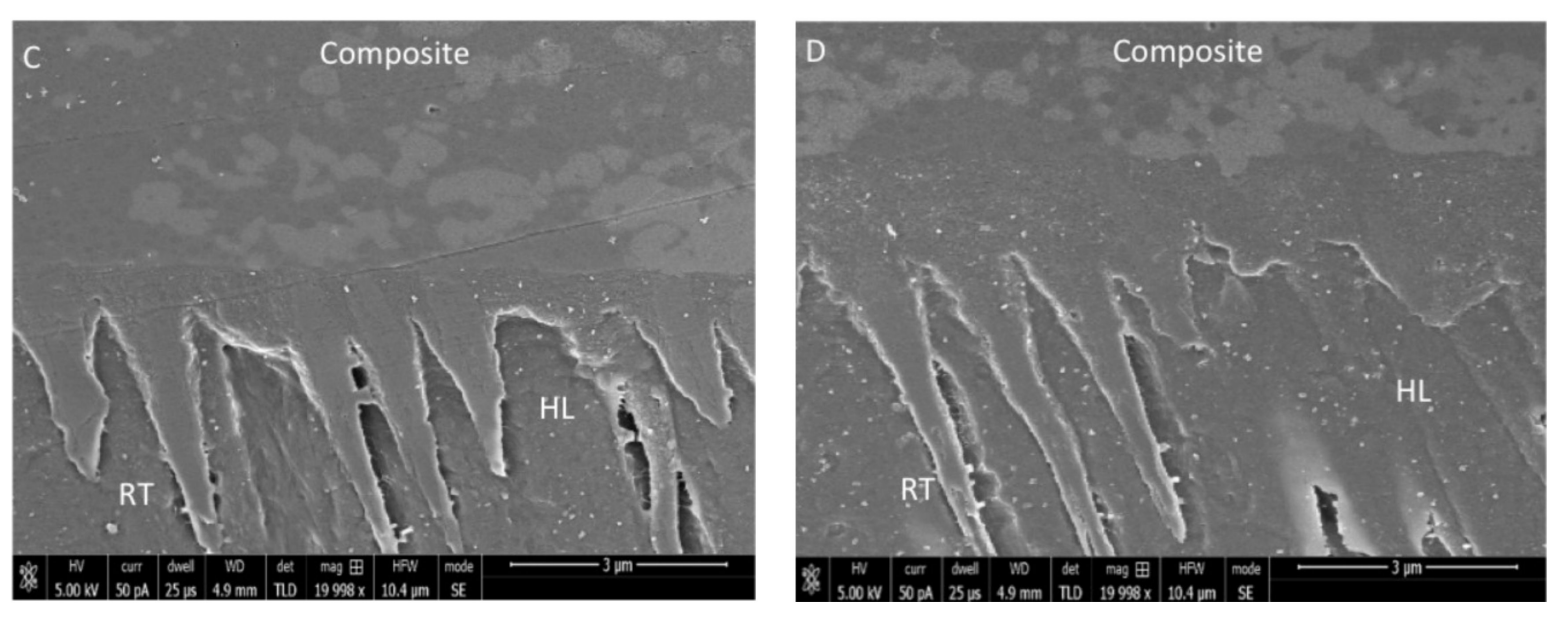
Resin–dentin-bonded samples using (**A**) unfilled adhesive (Si-EA-0%), (**B**) 5% silica-filled adhesive (Si-EA-5%), (**C**) 10% silica-filled adhesive (Si-EA-10%) and (**D**) 15% silica-filled adhesive (Si-EA-15%). The silica-filled and unfilled adhesive resins show similar resin tag formation, in contrast to high filler adhesive samples. (HL: hybrid layer, RT: resin tags).

The SEM analysis of Si particles before their incorporation in EA revealed 150nm sized non-porous particles that appeared to be agglomerated (Figure 1). The particles were spherical shaped devoid of any coarse edges. The resin–dentin interface demonstrated resin tag formation on SEM micrographs for gp-1 (Si-EA-0%) (Figure 2A), gp-2 (Si-EA-5%) (Figure 2B), gp-3 (Si-EA-10%) (Figure 2C), and gp-4 ((Si-EA-15%) (Figure 2D). It can be seen that in terms of resin tag formation, silica-filled adhesives showed similar or higher dentin penetration compared to unfilled adhesive (control) at the dentin interface.

**Figure 3:**
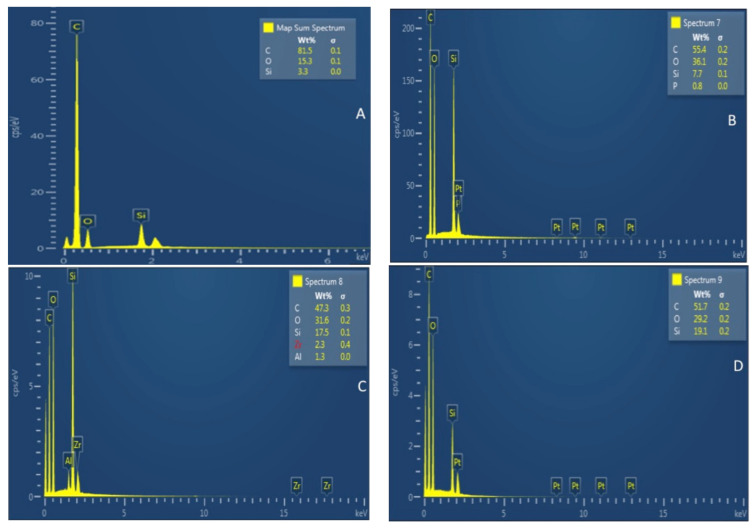
Energy dispersive X-ray spectroscopy (EDX) analysis of (**A**) unfilled adhesive (Si-EA-0%) (**B**) 5% silica-filled adhesive (Si-EA-5%) (**C**) 10% silica-filled adhesive (Si-EA-10%) and (**D**) 15% silica-filled adhesive (Si-EA-15%). Note the highest amount of silica (19.1%) in the 15% silica-filled adhesive as compared to the other groups with a low weight percentage of silica.

The EDX analysis of adhesives belonging to various groups revealed the presence of different elements. It can be seen that the greatest weight percentage (wt. %) of Si (19.1) was observed in gp-4 (Figure 3D), followed by gp-3 (17.1 Si wt. %) (Figure 3C). The next lowest wt. % of Si was seen in EDX mapping for gp-2 (7.7) (Figure 3B), whereas the lowest Si wt. % was appreciated for gp-1(3.3) (Figure 3A).

### 3.2. Micro-Raman Spectroscopy Results

**Figure 4:**
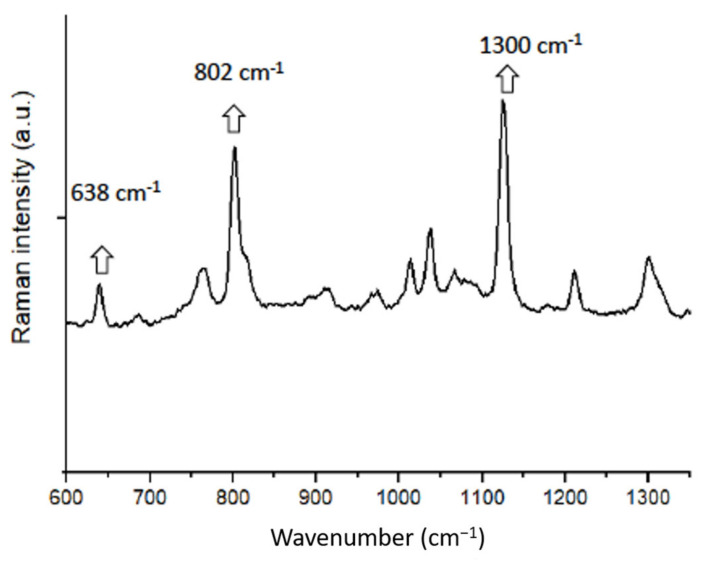
Micro-Raman analysis of silica powder at an excitation wavelength of 532 nm laser showed a spectrum having prominent peaks at 638, 802, and 1300 cm^−1^, characteristic of Si particles.

Micro-Raman analysis of silica powder at the excitation wavelength of 532 nm laser demonstrated a spectrum having distinctive peaks at 638, 802, and 1300 cm^−1^ (Figure 4). Among these, the strongest peak was observed at 1300 cm^−1^, followed by 802 and 638 cm^−1^. The presence of these three characteristic peaks shows the presence of Si particles in the sample [20].

### 3.3. µTBS and Failure Mode Analysis Results

The µTBS mean MPa values (Mean ± SD) observed for different adhesive groups in our study are presented in Table 1. It can be observed that the greatest µTBS values of non-aged and aged samples were observed for gp-4 (36.17 ± 3.31 and 29.77 ± 4.18, respectively) followed closely by gp-3 mean µTBS values (34.36 ± 4.23 and 27.07 ± 3.60 for non-aged and aged samples, respectively) (Table 1). The next lowest µTBS values were observed for gp-3, where non-aged samples had mean µTBS values of 28.85 ± 2.53 that were decreased after aging to 23.77 ± 2.49. The overall lowest mean µTBS values were seen for gp-1, where non-aged samples had mean µTBS values of 24.62 ± 2.53 that decreased to 20.77 ± 2.08. All the intra-group comparisons between non-aged and aged samples were statistically significant (*p* < 0.01). On the inter-group comparison for non-aged samples, statistically significant differences (*p* < 0.01) were seen when gp-1 and gp-2 were compared with all the other groups (Table 1). On inter-group comparison for aged samples, statistically significant differences (*p* < 0.01) were appreciated when gp-1 values were compared with gp-3 and gp-4 µTBS values and when gp-2 values were comparable to gp-3 and gp-4 values (Table 1).

Concerning failure mode analysis, no distinct pattern was observed; however, adhesive type failure was most common between all the adhesive groups (Table 1). The adhesive type failure is typically because of a failure in adhesion with fractures that are not seen in resin or the dentin [21]. In the present study, after adhesive type failures, mixed type failures were mostly observed, whereas cohesive type failures were only seen for gp-1 and gp-3 (Table 1).

### 3.4. FTIR and DC Analysis Results

**Figure 5:**
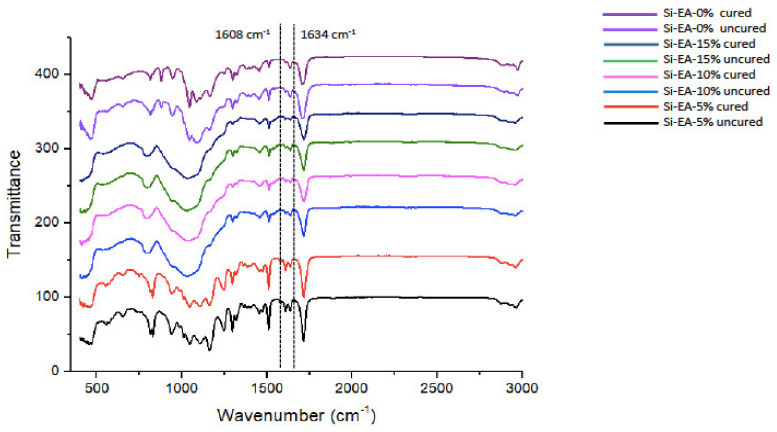
Fourier transform infrared (FTIR) spectrum of polymerized and un-polymerized unfilled adhesive (UA) and three test groups with 15%, 10% and 5% silica-experimental adhesive (Si-EA). The degree of conversion was calculated by estimating the changes in the peak height ratio of the absorbance intensities of the aliphatic C=C peak at 1638 cm^−1^ and that of an internal standard peak of aromatic C=C at 1608 cm^−1^ during polymerization, in relation to the uncured adhesive, as indicated by dotted lines.

For all the adhesive groups, representative FTIR spectra were recorded and merged together in Figure 5. The DC was computed by approximating the variations in the peak height ratio of the absorbance strengths of the aliphatic C=C peak at 1638 cm^−1^ and that of an inner standard peak of aromatic C=C at 1608 cm^−1^ during polymerization, as compared to the uncured adhesive, as indicated by dotted lines (Figure 5). The greatest mean DC was appreciated for gp-1 (48.2 ± 3.5) followed narrowly by gp-2 (47.4 ± 3.1). For gp-3, the mean DC was 41.9 ± 2.4, whereas the lowest mean DC was observed for gp-4 (37.6 ± 2.7) (Table 2). Statistically significant results (*p* < 0.01) were perceived when gp-1 and gp-2 values were compared with gp-3 and gp-4 DC values. No statistically significant differences were seen when the DC values of gp-1 were compared with gp-2, and when gp-3 values were matched with gp-4 (*p* > 0.01).

## 4. Discussion

Based on the findings of our study, the H_o_ was partly rejected, as Si inclusion in the EA led to an increase in the mean µTBS values; however, the DC of adhesive that included Si did not increase as compared with the control group. The key element controlling the success in adhesive dentistry is the bond strength [22]. Biodegradation of the adhesive interface over time is the main reason for decreased bond strength and adhesive interface failure [23]. Various previous studies have recommended the addition of filler particles to reduce degradation [24,25]. Si particles have previously been shown to facilitate the development of calcium-phosphate precursors (that is, a necessary step required for mineralization), thus performing as a nucleating mineral [26]. The presence of Si could also attract calcium particles to form a bioactive calcium silicate compound that can attach to phosphorus [27]. In order to obtain the benefits associated with silicate-based materials, we decided to include Si particles in different concentrations in our EA.

The Si particles that were commercially obtained for our study demonstrated a spherical shape on SEM micrographs (Figure 1). Filler particles that are spherical in shape could create a lubricating impact on the material, making it easily flowable with little effect on viscosity [28]. On the other hand, amorphous particles (without any clear shape) could impede the flow, resulting in an increased viscosity [28]. Resin tags are developed when the adhesive resin flows into the open dentinal tubules [29]. These resin tags ensure micromechanical interlocking by penetration in the tubules and formation of a stable hybrid layer [4]. The adhesives containing Si particles demonstrated comparable resin tags to the unfilled adhesive group. In our study, resin tags of variable depths were seen for all the adhesive groups (Figure 2A–D). However, the depth of the tags does not essentially affect the bond strength of the material, as demonstrated earlier by Anchieta et al. [30]. The EDX mapping of adhesives revealed the presence of various elements, the most important being Si. The highest wt. % of Si was observed for gp-4 and the lowest was seen for gp-1, demonstrating successful amalgamation of Si particles with the EA to formulate various adhesive groups.

The micro-Raman spectroscopy analysis revealed characteristic Si particle peaks observed at 638, 802, and 1300 cm^−1^. The differences in Raman spectra usually observed between 180 and 1300 cm^−1^ for silicate-based materials reflect the variable involvement of network-forming cations and network-modifying cations, which ultimately regulate the arrangement of bridging non-bridging oxygen in the silicate network [31]. Previously, Akram et al. demonstrated similar results and reported the presence of distinctive Raman peaks at 638, 802, and 1300 cm^−1^ [32], and our results are in conformity with their study.

The µTBS test values revealed the greatest bond strength for non-aged and aged gp-4 samples. A positive linear correlation between the increasing concentrations of Si particles and µTBS values was observed. This pattern clearly demonstrates that the addition of Si particles can increase the bond strength of the adhesives. Previously, Kasraei et al. demonstrated that the addition of 1% Si nanofiller resulted in increased bond strength as compared with the control group (without nanofiller addition) [33]. Kim et al. also reported that the addition of Si nanofiller in the adhesive resulted in the highest µTBS values compared with the other groups [34]. Fallahzadeh et al. reported similar findings and demonstrated that the addition of sepiolite (nanoparticles based on phyllosilicates) in the adhesive could improve its bond strength with dentin [35]. Our results are in conformity with these studies as we also observed that the addition of increasing concentrations of Si amplified the bond strength of the adhesives. We performed aging of the samples to mimic dynamic conditions faced by the materials in the oral cavity. According to the ISO standard 11405, thermal cycling of dental materials between 5 and 55 °C is appropriate to yield short-term aging [36]. Also, in an earlier study, it was suggested that 10,000 in vitro cycles of thermal cycling could reflect 1 year of in vivo service [37]. Therefore, our aging protocol could reflect 1 year of in vivo time. Previously, Helvatjoglu-Antoniades et al. reported a decreased bond strength of the adhesives when thermocycling was performed [38]. Our findings are in agreement with their results, as we also observed a decreased bond strength for all the adhesives after aging.

In the present study, the highest DC was achieved by gp-1 (unfilled adhesive) trailed narrowly by gp-2 (adhesives containing 5 wt. % Si particles). It was also observed that with the increasing Si concentration, DC values decreased linearly. Certain previous similar studies have also established that the increasing filler concentration may inversely impact the DC [10,39]. The probable reason for this finding could be that increased filler concentration provided a medium that was less favorable for adequate light penetration and conversion of monomers into polymers, thus causing less DC [10]. Additionally, increased concentration of filler could increase the viscosity of the material, triggering a consequential reduced DC [40], although this feature was not probed in our study.

The findings of our study, though revealing promising results, should be interpreted cautiously. We observed an improvement of µTBS values with an increasing concentration of Si particles; however, the DC also decreased linearly. The presence of a high DC is extremely desirable as it avoids the possibility of having a decreased interfacial strength [41]. Although we used 150nm sized Si particles, future studies with smaller Si particles are encouraged as the presence of smaller Si particles will ensure adequate polymerization by removing the presence of unreacted monomers, consequently leading to a high DC.

## 5. Conclusions

The incorporation of Si nanoparticles (10% and 15%) in polymer-based experimental dentin adhesive increased its bond strength as compared to unfilled adhesive resin. It also demonstrated suitable dentin interaction and improved resin tag formation (standard dentin hybrid layer). However, the addition of Si particles (10% and 15%) in adhesive decreased its degree of conversion compared to unfilled adhesive (0% Si—control).

## Figures and Tables

**Table 1:** Mean and SD of shear bond strength (SBS) and failure modes among the tested study groups.

	μTBS (MPa) (Mean ± SD)			Failure Mode Analysis (%)		
Group (*n* = 10)	Non-Aged	Aged	*p*-Value *	Adhesive	Cohesive	Mixed
1. Si-EA-0% (Control)	24.62 ± 2.53 ^a A^	-	<0.01	70	10	20
	-	20.77 ± 2.08 ^a B^		100	0	0
2. Si-EA-5%	28.85 ± 2.53 ^b A^			80	0	20
	-	23.77 ± 2.49 ^a B^		100	0	0
3. Si-EA-10%	34.36 ± 4.23 ^c A^			50	0	50
	-	27.07 ± 2.49 ^b B^		80	10	10
4. Si-EA-15%	36.17 ± 3.31 ^c A^			70	0	30
	-	29.97 ± 4.18 ^b B^		100	0	0

Si: Silica, EA: Experimental adhesive, μTBS: Micro-tensile bond strength, Mpa: Megapascal. Dissimilar lowercase letters within the same column denote a statistically significant difference (*p* < 0.01). Dissimilar capital letters in rows within the same group denote a statistically significant difference (*p* < 0.01). * ANOVA

**Table 2:** Means (SD) for the degree of conversion (%) values among study groups using ANOVA and the Tukey multiple comparisons test.

Groups		DC (Mean ± SD)	Tukey’s
1	Si-EA-0% (UA-Control)	48.2 ± 3.5	A
2	Si-EA-5%	47.4 ± 3.1	A
3	Si-EA-10%	41.9 ± 2.4	B
4	Si-EA-15%	37.6 ± 2.7	B

UA: Unfilled adhesive. Dissimilar capital letters denote a statistically significant difference among groups.

## Data Availability

The data is available on request from the corresponding author.
